# Letter to the Editor in response to “Find the real responders and improve the outcome of awake prone positioning”

**DOI:** 10.1186/s13054-021-03707-1

**Published:** 2021-08-04

**Authors:** Jacob Rosén, Erik von Oelreich, Diddi Fors, Malin Jonsson Fagerlund, Knut Taxbro, Peter Frykholm

**Affiliations:** 1 Department of Surgical Sciences, Section of Anaesthesiology and Intensive Care Medicine, Uppsala University, Entrance 78, 1 floor, 751 85 Uppsala, Sweden; 2 Perioperative Medicine and Intensive Care, Karolinska University Hospital, Solna, Sweden; 3 Department of Physiology and Pharmacology, Section of Anesthesiology and Intensive Care Medicine, Karolinska Institutet, Solna, Sweden; 4 Department of Anaesthesiology and Intensive Care Medicine, Ryhov County Hospital, Jönköping, Sweden

## Dear Editor,

We read with interest the letter by Wang and He [1] regarding our trial of awake prone positioning (APP) in severe COVID-19 [2] and would like to discuss the questions they raised.

First, Wang and He accurately point out that our study compares 3.4 h/day APP in the control group with 9.0 h/day APP in the intervention group. Since APP was frequently used even early in the pandemic, we found it unethical to prohibit APP in the control group when planning the trial. Furthermore, even though we would have liked to have seen > 16 h APP per day in the intervention group, only a minority of patients were able to reach this goal. Interestingly, the results were consistent in a subgroup of patients who received a median of 0.46 h/day of APP compared with patients who received 11.9 h/day of APP.

Second, we think there is still not enough evidence to state that APP decreases the risk of intubation. In the study by Ferrando et al. [3], duration of APP in the prone group was > 16 h/day which was much longer, not shorter as suggested by Wang and He, than in our study. In the study by Padrão et al. [4], intubation rates were higher in the group exposed to APP. The low overall intubation rate in our study could perhaps be explained by liberal use of non-invasive ventilation (NIV) [5] as well as a high level of care at the participating centers.

Third, Wang and He suggest that a majority of patients in our study were supported by NIV at randomization. On the contrary, 74% of patients in the control group and 86% of patients in the APP group had high-flow nasal oxygen (HFNO) at that point. However, a majority of our patients did receive NIV treatment during the trial for which initiation criteria were not protocolized. We could speculate that the possibility to switch between NIV and HFNO could be an advantage for some patients which may have influenced the results.

There are several unanswered questions regarding APP: when to start, the minimum effective dose per day, how to increase tolerance, if APP is more effective with HFNO or NIV alone or in combination. Large international trials as well as physiological studies are warranted to address these queries and ultimately to establish an evidence-based protocol for APP.

## Data Availability

Not applicable.

## Abbreviations

APP: Awake prone positioning
HFNO: High-flow nasal oxygen
NIV: Non-invasive ventilation

## References

[1] Wang H, He H (2021). Find the real responders and improve the outcome of awake prone positioning. Crit Care Lond Engl.

[2] Rosén J, von Oelreich E, Fors D, Jonsson Fagerlund M, Taxbro K, Skorup P (2021). Awake prone positioning in patients with hypoxemic respiratory failure due to COVID-19: the PROFLO multicenter randomized clinical trial. Crit Care.

[3] Ferrando C, Mellado-Artigas R, Gea A, Arruti E, Aldecoa C, Adalia R (2020). Awake prone positioning does not reduce the risk of intubation in COVID-19 treated with high-flow nasal oxygen therapy: a multicenter, adjusted cohort study. Crit Care.

[4] Padrão EMH, Valente FS, Besen BAMP, Rahhal H, Mesquita PS, de Alencar JCG (2020). Awake prone positioning in COVID-19 hypoxemic respiratory failure: exploratory findings in a single-center retrospective cohort study. Acad Emerg Med.

[5] Grieco DL, Menga LS, Cesarano M, Rosà T, Spadaro S, Bitondo MM (2021). Effect of helmet noninvasive ventilation vs high-flow nasal oxygen on days free of respiratory support in patients with COVID-19 and moderate to severe hypoxemic respiratory failure: the henivot randomized clinical trial. JAMA.

